# Supplementary material for: Evaluation of a group antenatal care intervention in two Northern Nigerian states: Quasi-experimental study
Source: PLoS One. 2025 Oct 8;20(10):e0333383. doi: 10.1371/journal.pone.0333383 (PMC12507231; doi:10.1371/journal.pone.0333383)
Supplement: S1 File — (DOCX) [file pone.0333383.s001.docx]

Supplementary Table S1. Associations between Baseline Characteristics and Loss-to-Follow-Up (LTFU) by State

|  | Kaduna |  | Kano |  |
| --- | --- | --- | --- | --- |
|  | LTFU % | p-value | LTFU % | p-value |
| Age Group |  |  |  |  |
| 15-19 | 17.5 | 0.917 | 6.4 | 0.934 |
| 20-24 | 18.8 |  | 6.9 |  |
| 25-29 | 20.2 |  | 6.1 |  |
| 30-34 | 19.8 |  | 5.3 |  |
| 35+ | 17.1 |  | 7.4 |  |
| Education |  |  |  |  |
| Never attended formal school | 21.2 | 0.934 | 5.4 | 0.908 |
| Primary | 17.0 |  | 7.1 |  |
| Secondary | 19.3 |  | 6.9 |  |
| Higher | 18.3 |  | 8.1 |  |
| Qur’anic/Islamiyya | 18.8 |  | 6.2 |  |
| Other | 25.0 |  | 0.0 |  |
| Employment |  |  |  |  |
| Unemployed | 21.4 | 0.188 | 7.0 | 0.769 |
| Employed | 15.9 |  | 5.9 |  |
| Own a business | 17.6 |  | 6.0 |  |
| Residence |  |  |  |  |
| Urban | 17.4 | 0.088 | 10.2 | 0.010 |
| Rural | 21.2 |  | 5.5 |  |
| Ever Given Birth Before |  |  |  |  |
| Yes | 18.6 | 0.520 | 6.4 | 0.928 |
| No | 20.1 |  | 6.5 |  |
| Number of Living Children |  |  |  |  |
| 0 | 22.2 | 0.677 | 6.5 | 0.175 |
| 1 | 19.6 |  | 9.3 |  |
| 2 | 19.8 |  | 8.1 |  |
| 3 | 16.4 |  | 3.5 |  |
| 4 | 14.2 |  | 3.7 |  |
| 5+ | 20.5 |  | 6.3 |  |
| Experienced Problems During Prior Pregnancy/Delivery* |  |  |  |  |
| Yes | 21.2 | 0.140 | 5.9 | 0.641 |
| No | 17.2 |  | 6.6 |  |
| Place of Delivery for Last Pregnancy* |  |  |  |  |
| Home | 18.2 | 0.992 | 4.8 | 0.009 |
| Facility | 18.5 |  | 8.1 |  |
| Other | 19.1 |  | 16.7 |  |
| Skilled Birth Attended at Last Delivery* |  |  |  |  |
| Yes | 17.0 | 0.064 | 7.2 | 0.225 |
| No or Don’t know | 22.1 |  | 5.3 |  |
| Postnatal Health Check within One Week of Last Delivery* |  |  |  |  |
| Yes | 16.3 | 0.000 | 6.5 | 0.798 |
| No or Don’t know | 29.2 |  | 6.0 |  |
| Any Postnatal Care Appointments after Last Delivery* |  |  |  |  |
| Yes | 17.1 | 0.004 | 5.9 | 0.187 |
| No or Don’t know | 28.3 |  | 8.6 |  |

*Analysis of associations with loss-to-follow-up for these variables is limited to participants who reported at least one prior pregnancy (n=960 in Kaduna and n=990 in Kano at baseline.

Supplementary Table S2. Coefficients and P-Values from the Multinomial Logistic Regression Model Predicting Number of gANC Meetings Attended (Reference Outcome: One gANC Session Attended)

|  | Equation for 0 vs 1 | | Equation for 2 vs 1 | | Equation for 3 vs 1 | | Equation for 4 vs 1 | | Equation for 5 vs 1 | |
| --- | --- | --- | --- | --- | --- | --- | --- | --- | --- | --- |
| Age Group (Ref: 15-19 years) | Coef. | p-value | Coef. | p-value | Coef. | p-value | Coef. | p-value | Coef. | p-value |
| 20-24 | 0.24 | (0.508) | 0.34 | (0.060) | 0.52 | (0.008) | 0.71 | (0.009) | 0.67 | (0.063) |
| 25-29 | 0.15 | (0.693) | -0.21 | (0.544) | 0.23 | (0.302) | 0.51 | (0.067) | 0.51 | (0.200) |
| 30-34 | 0.53 | (0.406) | -0.11 | (0.707) | 0.10 | (0.621) | 0.33 | (0.432) | 0.33 | (0.379) |
| 35+ | 0.24 | (0.687) | 0.02 | (0.955) | 0.14 | (0.681) | 0.38 | (0.375) | 0.50 | (0.313) |
| Education (Ref: Never attended) |  |  |  |  |  |  |  |  |  |  |
| Primary | 0.11 | (0.777) | 0.20 | (0.496) | -0.08 | (0.813) | -0.01 | (0.971) | 0.10 | (0.751) |
| Secondary | -0.15 | (0.729) | -0.05 | (0.864) | -0.33 | (0.250) | -0.12 | (0.603) | -0.02 | (0.949 |
| Qur’anic/Islamiyya | -0.04 | (0.931) | 0.04 | (0.929) | 0.07 | (0.899) | -0.15 | (0.735) | 0.38 | (0.473) |
| Other | 0.06 | (0.892) | 0.21 | (0.485) | -0.32 | (0.391) | -0.03 | (0.893) | -0.24 | (0.432) |
| Employment (Ref: Unemployed) |  |  |  |  |  |  |  |  |  |  |
| Employed | -0.69 | (0.145) | -0.74 | (0.273) | -0.31 | (0.522) | 0.16 | (0.761) | 0.22 | (0.632) |
| Own a Business | -0.04 | (0.743) | 0.30 | (0.124) | 0.41 | (0.003) | 0.36 | (0.025) | 0.45 | (0.004) |
| Residence (Ref: Urban) |  |  |  |  |  |  |  |  |  |  |
| Rural | -0.14 | (0.725) | 0.20 | (0.460) | 0.22 | (0.522) | 0.38 | (0.281) | 0.74 | (0.108) |
| Ever Given Birth Before (Ref: Yes) |  |  |  |  |  |  |  |  |  |  |
| No | -0.79 | (0.104) | -0.81 | (0.091) | -0.47 | (0.411) | -0.24 | (0.443) | -0.63 | (0.101) |
| Number of Living Children | -0.38 | (0.000) | -0.07 | (0.531) | -0.06 | (0.310) | -0.13 | (0.130) | -0.14 | (0.089) |
| State (Ref: Kaduna) |  |  |  |  |  |  |  |  |  |  |
| Kano | 1.72 | (0.000) | 0.16 | (0.280) | -0.50 | (0.102) | -0.47 | (0.169) | -1.14 | (0.012) |
| Experience Problems in Prior Pregnancy or Delivery (Ref: No Prior Pregnancy) |  |  |  |  |  |  |  |  |  |  |
| Yes | -0.20 | (0.744) | -0.98 | (0.091) | -0.80 | (0.377) | -0.84 | (0.288) | -1.44 | (0.035) |
| No | -0.64 | (0.321) | -1.24 | (0.029) | -0.74 | (0.412) | -0.77 | (0.344) | -1.39 | (0.056) |
| Place of Last Delivery (Ref: Home) |  |  |  |  |  |  |  |  |  |  |
| Facility | 1.07 | (0.033) | 0.66 | (0.335) | 0.44 | (0.616) | 0.47 | (0.459) | 0.71 | (0.322) |
| Other | 1.20 | (0.033) | 0.84 | (0.211) | 0.75 | (0.390) | 0.71 | (0.009) | 1.17 | (0.186) |
| Skilled Attendant and Last Delivery (Ref: No) |  |  |  |  |  |  |  |  |  |  |
| Yes | 0.05 | (0.869) | 0.25 | (0.195) | 0.35 | (0.032) | 0.51 | (0.067) | 0.19 | (0.306) |
| Postnatal Chack after Last Delivery (Ref: No) |  |  |  |  |  |  |  |  |  |  |
| Yes | 0.21 | (0.609) | -0.11 | (0.690) | -0.24 | (0.062) | 0.33 | (0.432) | 0.10 | (0.612) |
| Any Postnatal Care after Last Delivery (Ref: No) |  |  |  |  |  |  |  |  |  |  |
| Yes | -1.13 | (0.003) | -0.66 | (0.039) | -0.22 | (0.724) | 0.38 | (0.375) | -0.43 | (0.367) |

Supplementary Table S3. Raw and Inverse-Probability Weighted Standardized Differences between Women Attending Different Numbers of gANC Sessions

|  | 0 vs 1 Sessions | | 2 vs 1 Sessions | | 3 vs 1 Sessions | | 4 vs 1 Sessions | | 5 vs 1 Sessions | |
| --- | --- | --- | --- | --- | --- | --- | --- | --- | --- | --- |
| Age Group (Ref: 15-19 years) | Raw | Weighted | Raw | Weighted | Raw | Weighted | Raw | Weighted | Raw | Weighted |
| 20-24 | 0.20 | 0.13 | 0.23 | -0.06 | 0.19 | -0.04 | 0.25 | -0.05 | 0.21 | -0.04 |
| 25-29 | -0.09 | -0.04 | -0.17 | 0.00 | -0.01 | -0.01 | -0.01 | -0.00 | 0.02 | 0.00 |
| 30-34 | -0.03 | -0.06 | -0.10 | 0.03 | -0.08 | -0.01 | -0.12 | -0.00 | -0.11 | -0.01 |
| 35+ | -0.16 | 0.03 | -0.02 | 0.03 | -0.06 | 0.03 | -0.11 | 0.03 | -0.08 | 0.02 |
| Education (Ref: Never attended) |  |  |  |  |  |  |  |  |  |  |
| Primary | 0.05 | -0.23 | 0.08 | -0.02 | 0.02 | -0.01 | -0.00 | -0.01 | 0.01 | -0.01 |
| Secondary | -0.09 | 0.36 | -0.03 | 0.03 | -0.03 | 0.05 | 0.06 | 0.04 | 0.05 | 0.05 |
| Qur’anic/Islamiyya | -0.09 | -0.17 | -0.07 | -0.03 | 0.10 | -0.08 | 0.05 | -0.07 | 0.22 | -0.06 |
| Other | -0.02 | 0.09 | 0.03 | 0.04 | -0.06 | 0.04 | -0.01 | 0.03 | -0.08 | 0.03 |
| Employment (Ref: Unemployed) |  |  |  |  |  |  |  |  |  |  |
| Employed | -0.16 | -0.09 | -0.18 | 0.07 | -0.06 | 0.03 | 0.02 | 0.01 | 0.09 | 0.01 |
| Own a Business | -0.04 | 0.21 | 0.13 | -0.04 | 0.19 | -0.02 | 0.13 | -0.02 | 0.14 | 0.02 |
| Residence (Ref: Urban) |  |  |  |  |  |  |  |  |  |  |
| Rural | 0.21 | -0.01 | 0.12 | -0.06 | -0.05 | -0.01 | -0.01 | -0.01 | -0.04 | -0.04 |
| Ever Given Birth Before (Ref: Yes) |  |  |  |  |  |  |  |  |  |  |
| No | 0.04 | -0.13 | 0.07 | 0.00 | -0.05 | 0.04 | 0.06 | 0.02 | 0.02 | 0.01 |
| Number of Living Children | -0.28 | -0.01 | -0.13 | 0.02 | -0.09 | -0.03 | -0.21 | -0.03 | -0.23 | -0.03 |
| State (Ref: Kaduna) |  |  |  |  |  |  |  |  |  |  |
| Kano | 0.64 | -0.11 | 0.10 | -0.02 | -0.23 | -0.00 | -0.22 | 0.01 | -0.55 | 0.01 |
| Experience Problems in Prior Pregnancy or Delivery (Ref: No Prior Pregnancy) |  |  |  |  |  |  |  |  |  |  |
| Yes | 0.20 | 0.17 | 0.08 | 0.04 | -0.03 | -0.01 | -0.04 | 0.02 | -0.05 | 0.00 |
| No | -0.22 | -0.09 | -0.18 | -0.04 | 0.02 | -0.03 | -0.04 | -0.05 | -0.04 | -0.03 |
| Place of Last Delivery (Ref: Home) |  |  |  |  |  |  |  |  |  |  |
| Facility | 0.02 | -0.03 | -0.09 | -0.01 | -0.17 | -0.03 | -0.22 | -0.02 | -0.31 | -0.02 |
| Other | -0.03 | 0.10 | 0.03 | -0.00 | 0.22 | -0.02 | 0.19 | -0.02 | 0.29 | -0.01 |
| Skilled Attendant and Last Delivery (Ref: No) |  |  |  |  |  |  |  |  |  |  |
| Yes | -0.04 | 0.20 | 0.02 | 0.02 | 0.18 | -0.01 | 0.16 | -0.02 | 0.15 | -0.00 |
| Postnatal Chack after Last Delivery (Ref: No) |  |  |  |  |  |  |  |  |  |  |
| Yes | -0.10 | 0.09 | -0.13 | -0.03 | -0.00 | -0.07 | -0.02 | -0.04 | 0.01 | -0.02 |
| Any Postnatal Care after Last Delivery (Ref: No) |  |  |  |  |  |  |  |  |  |  |
| Yes | -0.23 | 0.12 | -0.17 | -0.05 | 0.01 | -0.07 | -0.08 | -0.05 | -0.04 | -0.04 |
