# Supplementary material for: Evaluation of a group antenatal care intervention in two Northern Nigerian states: Quasi-experimental study
Source: PLoS One. 2025 Oct 8;20(10):e0333383. doi: 10.1371/journal.pone.0333383 (PMC12507231; doi:10.1371/journal.pone.0333383)
Supplement: Attachment — Submitted filename: quasi study.pdf [file pone.0333383.s002.pdf]

PLOS ONE

Quasi-experimental evaluation of a Group Antenatal Care Intervention in Nigeria

--Manuscript Draft--

**Manuscript Number:**	PONE-D-25-15448
**Article Type:**	Research Article
**Full Title:**	Quasi-experimental evaluation of a Group Antenatal Care Intervention in Nigeria
**Short Title:**	Quasi-experimental evaluation of a Group Antenatal Care Intervention in Nigeria
**Corresponding Author:**	W. Douglas Evans, PhD<br>George Washington University School of Public Health and Health Services: The George Washington University Milken Institute of Public Health<br>Washington, District of Columbia UNITED STATES OF AMERICA
**Keywords:**	Women's and maternal health; Antenatal care; implementation science; quasi-experimental design; Nigeria; low and middle income country; healthcare facility; pregnancy
**Abstract:**	Background: In low- and middle-income countries (LMIC), group antenatal care (gANC) has the potential to reshape the traditional antenatal care (ANC) service delivery model, enhancing the care experience for both women and providers. ANC non-utilization rates are high in some parts of Nigeria, the evidence-based gANC program offers a structured yet flexible model that may increase maternal and child healthcare utilization which may in turn improve outcomes.<br>Methods: This is a longitudinal study of women who participated in the gANC program at healthcare facilities sampled in Kaduna and Kano states, Nigeria. We plan to follow this longitudinal cohort over 3 time points of 18 months and to use data on participation in the gANC program as an independent variable to predict program outcomes using multivariate analysis and propensity matching techniques. This is aimed at isolating the causal effect of the number of gANC meetings attended during the index pregnancy on the probability of delivering that pregnancy in a health facility. To achieve that objective, we used inverse-probability weighting in addition to adjusted multivariate logistic regression models.<br>Results: Overall, we found that there was high retention at follow-up, and generally high attendance at follow-up, with higher attendance based on other variables such as prior pregnancy and participants who were employed. We had low participants' attrition well within power analysis assumptions for the study. In terms of causal inference, there is a strong positive relationship between gANC session attendance and facility delivery, with women who attended five or more gANC sessions being approximately twice as likely to deliver in a health facility as those who attended none or one. Adjustment for a set of socio-demographic and prior pregnancy- and delivery-related variables via inverse probability weighting showed that a positive effect on facility delivery persists, especially at the highest levels of gANC session attendance.<br>Discussion: Results of this study tend to confirm the main findings from previous studies of gANC, which found that higher levels of participation resulted in higher facility delivery. This study found that it was both scalable in a diverse set of urban and rural healthcare facilities and engagement with the program among eligible women was high. Future research in the current longitudinal study will evaluate long-term effects of gANC on maternal and child health outcomes such as family planning and vaccination.<br>Conclusion: The gANC program at scale in Nigeria produced high levels of participation, and resulted in terms of facility birthing utilization were consistent with previous research. Future research should examine how to optimize program impact and enhance sustainability.
**Order of Authors:**	W. Douglas Evans, PhD
	Jeffrey B Bingenheimer, Ph.D.
	Taiseer Zaman
	Chinwe L Ochu
	Samson B Adebayo

Powered by Editorial Manager® and ProduXion Manager® from Aries Systems Corporation

	Fasiku A David
	Sani A Gar
	Masduk Abdulkarim
**Additional Information:**	
**Question**	**Response**
**Financial Disclosure**<br><br>Enter a financial disclosure statement that describes the sources of funding for the work included in this submission. Review the submission guidelines for detailed requirements. View published research articles from *PLOS ONE* for specific examples.<br><br>This statement is required for submission and **will appear in the published article** if the submission is accepted. Please make sure it is accurate.<br><br>**Funded studies**<br>Enter a statement with the following details:<br>• Initials of the authors who received each award<br>• Grant numbers awarded to each author<br>• The full name of each funder<br>• URL of each funder website<br>• Did the sponsors or funders play any role in the study design, data collection and analysis, decision to publish, or preparation of the manuscript?<br><br>Did you receive funding for this work?	Yes
Please add funding details.<br>as follow-up to "**Financial Disclosure**<br><br>Enter a financial disclosure statement that describes the sources of funding for the work included in this submission. Review the submission guidelines for detailed requirements. View published research articles from *PLOS ONE* for specific examples.<br><br>This statement is required for submission and **will appear in the published article** if	Gates Foundation grant INV-043363

the submission is accepted. Please make sure it is accepted.<br><br>**Funded studies**<br>Enter a statement with the following details:<br>• Initials of the authors who received each award<br>• Grant numbers awarded to each author<br>• The full name of each funder<br>• URL of each funder website<br>• Did the sponsors or funders play any role in the study design, data collection and analysis, decision to publish, or preparation of the manuscript?<br><br>Did you receive funding for this work?"	
Please select the country of your main research funder (please select carefully as in some cases this is used in fee calculation).<br>as follow-up to "**Financial Disclosure**<br><br>Enter a financial disclosure statement that describes the sources of funding for the work included in this submission. Review the submission guidelines for detailed requirements. View published research articles from *PLOS ONE* for specific examples.<br><br>This statement is required for submission and **will appear in the published article** if the submission is accepted. Please make sure it is accurate.<br><br>**Funded studies**<br>Enter a statement with the following details:<br>• Initials of the authors who received each award<br>• Grant numbers awarded to each author<br>• The full name of each funder<br>• URL of each funder website<br>• Did the sponsors or funders play any role in the study design, data collection and analysis, decision to publish, or preparation of the manuscript?	NIGERIA - NG

Powered by Editorial Manager® and ProduXion Manager® from Aries Systems Corporation

Did you receive funding for this work?"	
**Competing Interests**<br><br>Use the instructions below to enter a competing interest statement for this submission. On behalf of all authors, disclose any competing interests that could be perceived to bias this work—acknowledging all financial support and any other relevant financial or non-financial competing interests.<br><br>This statement is required for submission and **will appear in the published article** if the submission is accepted. Please make sure it is accurate and that any funding sources listed in your Funding Information later in the submission form are also declared in your Financial Disclosure statement.<br><br>View published research articles from *PLOS ONE* for specific examples.<br><br>**NO authors have competing interests**<br><br>Enter: *The authors have declared that no competing interests exist.*<br><br>**Authors with competing interests**<br><br>Enter competing interest details beginning with this statement:<br><br>*I have read the journal's policy and the authors of this manuscript have the following competing interests: [insert competing interests here]*<br><br>* typeset	The authors have declared that no competing interests exist.
**Ethics Statement**<br><br>Enter an ethics statement for this submission. This statement is required if the study involved:<br><br>• Human participants<br>• Human specimens or tissue	Ethical approval was obtained from the Nigeria’s National Health Research Ethics Committee (NHREC) under approval number NHREC/01/01/2007-12/01/2024. An ethical waiver was obtained from the Institutional Research Board (IRB) of The George Washington University. Only participants who gave informed consent were enrolled into the study. Informed consent was obtained verbally based on an IRB-approved statement read by a trained interviewer and witnessed by a second interviewer and recorded. Privacy and confidentiality of data were maintained throughout data collection, storage, and analysis.

• Vertebrate animals or cephalopods<br>• Vertebrate embryos or tissues<br>• Field research<br><br>Write "N/A" if the submission does not require an ethics statement.<br><br>General guidance is provided below. Consult the submission guidelines for detailed instructions. **Make sure that all information entered here is included in the Methods section of the manuscript.**	

Powered by Editorial Manager® and ProduXion Manager® from Aries Systems Corporation

**Format for specific study types**<br><br>**Human Subject Research (involving human participants and/or tissue)**<br>• Give the name of the institutional review board or ethics committee that approved the study<br>• Include the approval number and/or a statement indicating approval of this research<br>• Indicate the form of consent obtained (written/oral) or the reason that consent was not obtained (e.g. the data were analyzed anonymously)<br><br>**Animal Research (involving vertebrate animals, embryos or tissues)**<br>• Provide the name of the Institutional Animal Care and Use Committee (IACUC) or other relevant ethics board that reviewed the study protocol, and indicate whether they approved this research or granted a formal waiver of ethical approval<br>• Include an approval number if one was obtained<br>• If the study involved *non-human primates*, add *additional details* about animal welfare and steps taken to ameliorate suffering<br>• If anesthesia, euthanasia, or any kind of animal sacrifice is part of the study, include briefly which substances and/or methods were applied<br><br>**Field Research**<br><br>Include the following details if this study involves the collection of plant, animal, or other materials from a natural setting:<br>• Field permit number<br>• Name of the institution or relevant body that granted permission	
**Data Availability**<br><br>Authors are required to make all data underlying the findings described fully available, without restriction, and from the time of publication. PLOS allows rare exceptions to address legal and ethical concerns. See the PLOS Data Policy and FAQ for detailed information.	No - some restrictions will apply

Powered by Editorial Manager® and ProduXion Manager® from Aries Systems Corporation

A Data Availability Statement describing where the data can be found is required at submission. Your answers to this question constitute the Data Availability Statement and **will be published in the article**, if accepted.<br><br>**Important:** Stating 'data available on request from the author' is not sufficient. If your data are only available upon request, select 'No' for the first question and explain your exceptional situation in the text box.<br><br>Do the authors confirm that all data underlying the findings described in their manuscript are fully available without restriction?	
**Describe where the data may be found in full sentences. If you are copying our sample text, replace any instances of XXX with the appropriate details.**<br><br>• If the data are **held or will be held in a public repository**, include URLs, accession numbers or DOIs. If this information will only be available after acceptance, indicate this by ticking the box below. For example: *All XXX files are available from the XXX database (accession number(s) XXX, XXX.).*<br>• If the data are all contained **within the manuscript and/or Supporting Information files**, enter the following: *All relevant data are within the manuscript and its Supporting Information files.*<br>• If neither of these applies but you are able to provide **details of access elsewhere**, with or without limitations, please do so. For example:<br><br>*Data cannot be shared publicly because of [XXX]. Data are available from the XXX Institutional Data Access / Ethics Committee (contact via XXX) for researchers who meet the criteria for access to confidential data.*<br><br>*The data underlying the results presented in the study are available from (include the name of the third party*	The data will be made available upon a reasonable written request to the corresponding author.

and contact information or URL).<br>• This text is appropriate if the data are owned by a third party and authors do not have permission to share the data.<br><br>* typeset	
Additional data availability information:	

Powered by Editorial Manager® and ProduXion Manager® from Aries Systems Corporation

Manuscript Click here to access/download;Manuscript;gANC_midline_ms_submit.docx

Quasi-experimental evaluation of a Group Antenatal Care Intervention in Nigeria

W. Douglas Evans[1], Jeffrey B. Bingenheimer[1], Taiseer Zaman[1], Chinwe L. Ochu[1], Samson Babatunde Adebayo[2], Fasiku Adekunle David[2], Sani Ali Gar[2], and Masduk Abdulkarim[3]

Authors' Affiliations

[1] Milken Institute School of Public Health, The George Washington University, 950 New Hampshire Avenue NW, Washington, DC 20052, USA

[2] Data Research and Mapping Consults, Suite: B 14, Dansarari Plaza, 5 Ziguinchor Str, Wuse Zone 4, Abuja 900284, Nigeria

[3] Gates Foundation, Nigeria Country Office, Global Development Division, 45 Aguiyi Ironsi St, Wuse, Abuja 904101, Nigeria

Corresponding Author:

W. Douglas Evans

Professor, Department of Prevention and Community Health & Global Health

Milken Institute School of Public Health

The George Washington University

wdevans@gwu.edu

Abstract

Background: In low- and middle-income countries (LMIC), group antenatal care (gANC) has the potential to reshape the traditional antenatal care (ANC) service delivery model, enhancing the care experience for both women and providers. ANC non-utilization rates are high in some parts of Nigeria, the evidence-based gANC program offers a structured yet flexible model that may increase maternal and child healthcare utilization which may in turn improve outcomes.

Methods: This is a longitudinal study of women who participated in the gANC program at healthcare facilities sampled in Kaduna and Kano states, Nigeria. We plan to follow this longitudinal cohort over 3 time points of 18 months and to use data on participation in the gANC program as an independent variable to predict program outcomes using multivariate analysis and propensity matching techniques. This is aimed at isolating the causal effect of the number of gANC meetings attended during the index pregnancy on the probability of delivering that pregnancy in a health facility. To achieve that objective, we used inverse-probability weighting in addition to adjusted multivariate logistic regression models.

Results: Overall, we found that there was high retention at follow-up, and generally high attendance at follow-up, with higher attendance based on other variables such as prior pregnancy and participants who were employed. We had low participants' attrition well within power analysis assumptions for the study. In terms of causal inference, there is a strong positive relationship between gANC session attendance and facility delivery, with women who attended five or more gANC sessions being approximately twice as likely to deliver in a health facility as those who attended none or one. Adjustment for a set of socio-demographic and prior pregnancy- and delivery-related variables via inverse probability weighting showed that a positive effect on facility delivery persists, especially at the highest levels of gANC session attendance.

Discussion: Results of this study tend to confirm the main findings from previous studies of gANC, which found that higher levels of participation resulted in higher facility delivery. This study found that it was both scalable in a diverse set of urban and rural healthcare facilities and engagement with the program among eligible women was high. Future research in the current longitudinal study will evaluate long-term effects of gANC on maternal and child health outcomes such as family planning and vaccination.

Conclusion: The gANC program at scale in Nigeria produced high levels of participation, and resulted in terms of facility birthing utilization were consistent with previous research. Future research should examine how to optimize program impact and enhance sustainability.

Word counts:

Abstract: 420

Main Text: 4326

Introduction

In low- and middle-income countries (LMIC), group antenatal care (gANC) has the potential to reshape the traditional antenatal care (ANC) service delivery model, enhancing the care experience for both women and providers.[1] In Nigeria, some regions in the north of the country show ANC non-utilization rates of over 50%.[2] Research is needed to understand how to overcome barriers to, and increase utilization of ANC in LMIC, especially among underserved regions of countries such as Nigeria.

Financial, access, and other barriers limit participation in antenatal care.[3] Moreover, there is no strong evidence that standard antenatal education improves birth or parenthood outcomes. In contrast, gANC offers a structured yet flexible model that integrates key components, including continuity of midwifery care, social support, and inquiry-based learning, each of which may have distinct mechanisms of impact. A systematic realist review identified six interconnected mechanisms through which gANC can enhance maternal care experiences: social support, peer learning, active health participation, health education, and satisfaction or engagement with care.[4] Additionally, shared health activities and women-led, group-based discussions have been shown to foster more equal and trusting relationships between women and midwives.[5,6]

One critical area where gANC may provide significant benefits is in improving maternal knowledge and health behaviors. Studies indicate that maternal awareness of newborn danger signs and optimal newborn care practices is generally low. However, recognizing neonatal danger signs after birth strongly predicts care-seeking behavior for newborn illnesses. A study assessing the impact of gANC found that women in the intervention group had significantly higher knowledge of newborn danger signs and healthy newborn practices over time compared to those receiving individual ANC (I-ANC).[7]

Birth preparedness and complication readiness (BPCR) is another crucial factor influencing maternal and neonatal outcomes.[8] Limited BPCR has been identified as a major contributor to persistently high maternal and neonatal morbidity and mortality in low- and middle-income countries. BPCR includes raising awareness of danger signs, improving problem recognition, reducing delays in seeking care, choosing a birth location and provider in advance, knowing the nearest skilled provider, obtaining basic safe birth supplies, and identifying someone to accompany the woman to the facility during labor. A study found that BPCR scores significantly increased in the gANC group compared to the I-ANC group, suggesting that this model enhances women's preparedness for childbirth and emergencies.

The feasibility and acceptability of gANC have been evaluated in various settings. In Asia, particularly in India, a study explored the potential implementation of gANC, incorporating a clinical care component.[9] Stakeholders' perceptions, assessed through qualitative research, indicated strong support for the model following participation in a mock session demonstration. Women in gANC were also more likely to take prenatal vitamins and iron, use contraception at two months postpartum, and report higher satisfaction with the information provided, their relationship with healthcare providers, and overall quality of care.

Evidence from Ghana further supports the benefits of gANC. A cluster-randomized controlled trial comparing gANC with standard care found that by 34 weeks of gestation, women in the intervention group had significantly greater knowledge of family planning (FP) methods and a stronger intention to use FP after childbirth.[10] FP uptake was also consistently higher in the intervention group at all post-birth time points, except at 11–14 months postpartum, where the control group had higher rates.

Beyond family planning, gANC may also contribute to improved maternal health outcomes by increasing the uptake of key malaria in pregnancy (MIP) interventions. Malaria in pregnancy accounts for approximately 20% of stillbirths in sub-Saharan Africa and causes 10,000 maternal deaths worldwide each year.[11] A study conducted in Nigeria and Kenya suggests that gANC may enhance the uptake of intermittent preventive treatment in pregnancy with sulfadoxine-pyrimethamine (IPTp-SP), likely due to improved ANC retention.[12] Women in the intervention group had significantly higher IPTp-SP uptake compared to those receiving I-ANC (Nigeria: 3.45 vs. 2.14, $p < 0.001$; Kenya: 3.81 vs. 2.72, $p < 0.001$).

Overall, gANC presents a promising alternative to conventional antenatal care by integrating education, peer support, and active health engagement. Its ability to improve maternal knowledge, preparedness for birth, postnatal care utilization, and uptake of key maternal health interventions highlights its potential to transform ANC service delivery, particularly in resource-limited settings.

The present study reports on a follow-up (midline) of a large-scale gANC implementation project in Nigeria. The first phase (i.e. the baseline) of the study was implemented in Kaduna and Kano states in Nigeria in March and April 2024.[13] The second phase of the project, follow-up data collection from the longitudinal cohort of women recruited in the first phase, was implemented in Kaduna and Kano in October and November 2024. This study reports on analysis of the follow-up midline data from the second phase of the project. A future study will report on final findings from the project during the endline evaluation.

The key research questions addressed in this second midline phase of the project addressed implementation and primary effectiveness of gANC in promoting healthcare facility utilization. For instance, we asked: 1) To what extent was the gANC model implemented in Kaduna and

Kano, how successful was the study in following women recruited in the first phase? 2) To what extent did attending more gANC sessions make women in our sample more likely to deliver in a health facility.

METHODS

Study Design

The full study design has been described in a previous publication.[13] Briefly, this is a longitudinal study of women who participated in the gANC program at healthcare facilities sampled in Kaduna and Kano states, Nigeria. We plan to follow this longitudinal cohort over 3 time points of 18 months and to use data on participation in the gANC program as an independent variable to predict program outcomes using multivariate analysis and analytic matching techniques.[14]

This paper focuses on changes in key variables among the longitudinal sample from baseline to midline follow-up data. The main outcome of interest is delivery of the index pregnancy measured at baseline in a healthcare facility among women who had some level of participation in the gANC program. The third and final wave (i.e. endline) of the longitudinal study will examine measures of utilization of and satisfaction with care for gANC services over the course of the pregnancy, as well as perinatal outcomes including facility delivery, completion of postnatal health checks, postpartum family planning use, and uptake of recommended child immunizations.

Intervention

The gANC intervention has been described in detail in previous publications.[13,15] Briefly, the intervention consists of which includes group social support and ANC counseling, which are

theorized to increase satisfaction with and intention to utilize healthcare facilities for prenatal care and birthing.[15] This study is underpinned by the Diffusion of Innovation theory that explores how gANC activities are adopted and becomes a standard of care in northern Nigeria.[16] This model provides a basis for the conceptual framework of the program, which posits that ANC utilization can be influenced to promote the adoption of change, which in this case is the gANC intervention.[17] Observing the implementation of gANC in real life can provide important insight into facilitators and barriers that should be considered in planning for the scaling of the intervention in the States.[18]

Data Collection

Baseline data collection for this study was conducted in Kaduna and Kano States from 25 March to 8 April of 2024. The follow-up midline data collection was conducted in these same locations from 27 October to 20 November of 2024.

A 154-item Questionnaire was used for the quantitative midline survey. The survey tool was pilot-tested prior to implementation. Data collection was conducted during 15 days of fieldwork in October and November 2024. Eligible women (age 15 – 49 years at 12 - 20 weeks GA) who were recruited from randomly selected sample of facilities in each state at baseline were recontacted at follow-up. In each state, the quantitative survey was composed of three broad teams of interviewers and supervisors, one team per senatorial district. In each senatorial district, 10 interviewers and two supervisors conducted fieldwork.

Measures

The focal independent variable is number of gANC meetings attended during the index pregnancy. This was assessed by a series of questions in the follow-up questionnaire. The first

question read, “Have you ever attended any group ANC meetings?” Those who answered affirmatively were then asked about their attendance at each of five gANC sessions: (1) preventing problems during pregnancy; (2) recognizing problems during pregnancy and joint planning on topics for male engagement; (3) birth preparedness, complication readiness, and health timing and spacing of pregnancies; (4) review of individual plans and family planning intentions; and (5) what to expect in labor and birth, recognizing postpartum problems, exclusive breastfeeding, and final birth planning. Using responses to these questions we computed the total number of gANC meetings attended by each participant, ranging from zero to five.

The focal dependent variable is delivery of the index pregnancy at a health facility (as opposed to at home or elsewhere). This was assessed by a series of two questions in the follow-up questionnaire. First, participants were asked, “Did you identify a facility for the delivery of your pregnancy?” Those who answered affirmatively were then asked, “Did you deliver at the facility you identified?” Based on responses to these questions we classified participants as having delivered the index pregnancy in a health facility, or not.

Our analysis also makes use of twelve covariates that were assessed at baseline. Five of these were basic sociodemographic variables: state of residence, urban versus rural residence, age, education, and employment. The remaining seven focused on aspects of prior pregnancy, delivery, and postpartum experiences. All participants were asked whether they had ever had a previous live birth, and how many living children they had. Those who had at least one prior live birth were asked whether they experienced problems in their most recent prior pregnancy, whether they delivered that pregnancy in a health facility, whether the delivery included a skilled birth attendant, whether they received a postnatal health check within one week following the delivery, and whether they received any postnatal health checks following that delivery.

Data Analysis

Analysis of the data proceeded as follows. After merging the baseline and follow-up data, we first examined attrition and retention. We obtained the number and percentage of baseline participants who were lost to follow-up and tabulated the reasons for attrition as reported by data collection personnel. We then examined patterns of attrition by cross-tabulating baseline socio-demographic variables (age, education, employment, urban vs rural residence, and parity); and, among those who had given birth at least once prior to the index pregnancy, cross-tabulating variables characterizing participants' most recent pregnancy, birth, and postpartum experiences. We used chi-squared tests of independence to determine whether levels of attrition were statistically significantly different across levels of those baseline sociodemographic and prior pregnancy variables.

Next, we examined participation in gANC meetings (our focal independent variable) among participants whose index pregnancy had ended in a live birth prior to the follow-up interview. We obtained the count and percent of participants who attended each of five gANC meetings; computed the total number of gANC meetings each participant had attended; obtained counts and percents of the number of participants attending zero, one, two, three, four, and five gANC sessions; and obtained the mean number of gANC meetings attended. We then examined patterns of gANC meeting attendance by comparing the mean number of sessions attended across categories of baseline socio-demographic variables (age, education, employment, urban vs rural residence, and parity); and, among those who had given birth at least once prior to the index pregnancy, by comparing the mean number of gANC sessions attended across values of variables characterizing participants' most recent pregnancy, birth, and postpartum experiences. We used one-way analysis of variance to determine whether the mean number of sessions attended was

statistically significantly different across levels of these baseline sociodemographic and prior pregnancy variables.

We then turned our attention to the primary endpoint: facility delivery. Among participants whose index pregnancy had ended in a live birth prior to the follow-up interview, we obtained the count and percent who reported delivering the index pregnancy in a health facility. We then examined whether the proportion of participants whose delivery occurred in a health facility varied in relation to baseline socio-demographic variables (age, education, employment, urban vs rural residence, and parity); and, among those who had given birth at least once prior to the index pregnancy, by values of variables characterizing participants' most recent prior pregnancy, birth, and postpartum experiences. These analyses were conducted via cross-tabulations, and we use chi-squared tests of independence to determine whether variations in the prevalence of facility delivery across socio-demographic and prior pregnancy variables were statistically significant.

Finally, we carried out a series of analyses to answer our main research question: Whether attending more gANC sessions makes women more likely to deliver in a health facility. We first examine whether the proportion delivering in a health facility varies in relation to number of gANC meetings attended by cross-tabulating those two variables and carrying out a chi-squared test of independence. We then run two logistic regression models, both with facility delivery as the dependent variable and number of gANC sessions attended as the focal independent variable. As a measure of model building, our first model had no control variables; the second model controlled for baseline sociodemographic variables. The purpose of this analysis is to determine whether the association between gANC meeting attendance and facility delivery is independent of potential confounding variables.

The objective of this study is to isolate the causal effect of number of gANC meetings attended during the index pregnancy on the probability of delivering that pregnancy in a health facility. To achieve that objective, we used a further analytic approach called inverse-probability weighting.[19,20] This approach is similar to propensity score methods in that a statistical model is used to collapse all of the information in the entire set of covariates relevant to treatment assignment into a single number. Inverse-probability weighting uses the inverse of the propensity score as a weight for treated participants, and the inverse of the complement of the propensity score as a weight for untreated participants. This approach generalizes to independent variables, like number of gANC meetings attended, that take more than two values. A multinomial logistic regression model is used to relate the set of covariates to the number of gANC visits, and from this model we obtain for each participant the predicted probability the number of gANC sessions that she attended. The inverse of this predicted probability is then used as a weight in a model (in this case, logistic regression) relating the gANC meetings attended to the outcome of facility delivery. As a point of comparison, we also ran a more conventional regression-adjusted approach via a logistic regression model with facility delivery as the dependent variable, and number of gANC meetings attended and all twelve covariates as the independent variables.

Prior to running the inverse-probability weighted regression analysis, we first characterize the sample in terms of the distribution of all baseline covariates, number of gANC meetings attended, and facility delivery using simple frequencies. We next examine the associations between each of the twelve covariates and number of gANC meetings attended by obtaining the mean and standard deviation of number of sessions attended within each level of each covariate, and by running linear regressions in which each covariate predicts number of sessions attended. Following this, we examine the associations between the twelve covariates and facility delivery.

In the linear and logistic regression models described here, we use cluster rather than conventional standard errors, with local government authority as the clustering variable, in order to obtain p-values that accurately reflect the study's complex sampling design. We use the same approach of clustered standard errors in our inverse-probability weighting analyses. All data management and analyses were carried out in Stata 18.0.[21] For the inverse-probability weighting analysis we use Stata's *teffects ipwra* command.

Research Ethics

Ethical approval was obtained from the Nigeria's National Health Research Ethics Committee (NHREC). An ethical waiver was obtained from the Institutional Research Board (IRB) of The George Washington University. Only participants who gave informed consent were enrolled into the study. Informed consent was obtained verbally based on an IRB-approved statement read by a trained interviewer and witnessed by a second interviewer and recorded. Privacy and confidentiality of data were maintained throughout data collection, storage, and analysis.

RESULTS

Of the original sample of 2,469 pregnant women, 2,151 (87.1%) were located and reinterviewed at follow-up. Of the 318 who were lost to follow-up, most (117, 71.4%) had moved away from the study area, while some (21, 6.6%) had died, and the remainder (70, 22.0%) refused to participate or were otherwise unavailable at follow-up throughout the data collection period. Also excluded from the analysis are 199 who had lost their pregnancies, and 12 who were still pregnant with the index pregnancy at the time of follow-up.

Table 1 shows the composition of the analytic sample of n=1,952 in terms of socio-demographic background and aspects of prior pregnancy experiences. The sample is close to evenly

distributed between the two study states of Kaduna and Kano, and is heterogeneous with respect to age, education, employment status, and number of living children. Participants were more likely to reside in rural areas than in urban areas, and just over three-quarters had given birth before. With respect to prior pregnancy experiences, about one-quarter had experienced problems in their most recent prior pregnancy, and approximately equal numbers had delivered their most recent pregnancy in a health facility as opposed to at home. Most prior deliveries involved the presence of a skilled birth attendant, although a substantial minority occurred without one. Most reported having had a postnatal health check within one week of their most recent delivery, and an even larger fraction had at least one postnatal care appointment following that delivery.

[TABLE 1]

Table 2 shows the distribution of the focal independent and dependent variables: number of gANC meetings attended during the index pregnancy, and whether the index pregnancy was delivered in a health facility or not, respectively. In terms of number of gANC sessions attended, the distribution is skewed toward higher values, with five sessions being the modal value, followed by four sessions, and so on. A non-trivial proportion of just under 10% reported attending no gANC sessions at all during the index pregnancy. Just under half of the participants reported delivering the index pregnancy in a health facility.

[TABLE 2]

Associations between the covariates and number of gANC meetings attended during the index pregnancy are shown in Table 3. The average number of meetings attended by study participants was 3.23, and this varied significantly across the states, with the average participant in Kaduna

attending nearly an entire additional meeting than the average participant in Kano (p=0.012). None of the differences across levels of the other variables were statistically significant, but some were substantial in magnitude and came close to achieving statistical significance. Among those who had at least one prior delivery, for example, those who had their most recent delivery at a health facility attended an average of 3.45 gANC meetings during the index pregnancy, while those who delivered at home attended an average of 3.00. Likewise, those who reported attending a postnatal health check within one week of their prior delivery attended an average of 3.27 gANC meetings during the current pregnancy, while those who did not attended an average of just 2.94. The participants who reported being employed at baseline attended an average of 3.77 gANC meetings during the index pregnancy, compared to 3.06 among those who were not employed and an intermediate average of 3.30 among those employed in a self- or family-owned business. Together, these findings suggest that these covariates influence the number of gANC meetings attended to a modest degree.

[TABLE 3]

Table 4 shows associations between the covariates and facility delivery. In general, these associations are more substantial than those between the covariates and number of gANC sessions attended, and almost all of the associations are statistically significant. For example, facility delivery was almost twice as common in Kaduna (64.0%) as in Kano (34.6%), and was positively associated with higher education, urban residence, and employment status. Facility delivery was more common among those who had never given birth before; and was negatively associated with the number of living children. Facility delivery was also positively associated with several indicators of health care utilization during prior pregnancy, including facility

delivery during that pregnancy, the presence of a skilled birth attendant, and receipt of post-natal care.

[TABLE 4]

The unadjusted and adjusted associations between number of gANC meetings attended during the index pregnancy and facility delivery are shown in Table 5. The percentage delivering in a health facility increases consistently from as low as 28.3% among those who attended no gANC meetings, to as high as 61.4% among those who attended five or more meetings. The statistical significance of these unadjusted differences is shown in the "Unadjusted" panel. While the prevalence of facility delivery was not statistically distinguishable among those who attended no meetings or those who attended two meetings compared to those who attended only one, the odds of facility delivery were substantially higher among those attending three, four, or five or more meetings. That basic pattern remains when conventional regression adjustment is used to address confounding, as shown in the "Regression Adjusted" panel of Table 5. While the adjusted odds ratios for three, four, and five meetings are somewhat closer to the null than in the unadjusted model, all three remain positive and statistically significant. Much more substantial attenuation in the association between gANC meeting attendance and facility delivery when inverse probability weighting is used to address confounding. In that analysis, the effect of attending three as opposed to one meeting is no longer statistically significant. The effects of attending four or five or more gANC meetings remain statistically significant, but are much smaller in magnitude.

[TABLE 5]

DISCUSSION

In this study, we examined: 1) implementation of gANC intervention and longitudinal follow-up of women who participated in gANC, and 2) whether attending a larger number of gANC sessions increased the likelihood of facility delivery among pregnant women in Kaduna and Kano states in Nigeria.

In terms of the first study aim, we found that there was high retention at follow-up (87.1%), and generally high attendance at follow-up (3.23 gANC meetings), with higher attendance based on other variables such as prior pregnancy and participants who were employed. In addition, participation rates skewed to the high end of the scale of possible meetings attended, with the modal participant rate estimated at 5 gANC meetings, and next highest being 4 meetings. Overall, gANC implementation proceeded as planned, and consistent with prior studies,[14] and our attrition rate was lower than projected for power analysis.

In terms of the second aim, there is a strong positive relationship between gANC session attendance and facility delivery, with women who attended give or more gANC sessions being approximately twice as likely to deliver in a health facility as those who attended none or one. Adjustment for a set of socio-demographic and prior pregnancy- and delivery-related variables via inverse probability weighting, however, substantially attenuates that association. Evidence of a positive effect on facility delivery persists, especially at the highest levels of gANC session attendance. It is evident, however, that simple or even conventional regression-adjusted estimates substantially overstate the impact of gANC meeting attendance on facility delivery.

Overall, results of this study tend to confirm the main findings from previous studies of gANC, which found that higher levels of participation resulted in higher facility delivery.[15] This study examined implementation of gANC at scale in 2 large Nigerian states, and found that it is both scalable in a diverse set of urban and rural healthcare facilities, and engagement (in terms of

meeting attendance) with the program among eligible women was high. The program is a promising strategy to promote maternal and child health in LMICs.[22]

Limitations

Two main limitations of this study should be noted. First, both the focal independent variable of number of gANC meetings attended, as well as the dependent variable of facility delivery of the index pregnancy, were assessed via participant self-report. This could lead to upward or downward bias in our observed associations between gANC meeting attendance and facility delivery. Both gANC meeting attendance and facility delivery may be subject to some degree of social desirability bias, and some participants may be more inclined than others to provide socially desirable responses.

The second main limitation is the possibility of omitted variable bias. While we had a substantial set of covariates that included both sociodemographic background variables as well as characteristics of participants' previous pregnancy, delivery, and postpartum experiences, other confounders could have been omitted from this set. This concern may be mitigated to some extent by the associations between included covariates and omitted confounders. One possible confounder that was not included in our analysis, for example, was the distance between each participant's dwelling and the health facility, or more generally the difficulty involved in traveling from the dwelling to the facility. Such omitted variable bias may have affected results of the inverse-probability weighted analyses.

Future Research

This is the second in a series of three longitudinal studies on the gANC program in Nigeria. Future research in this study at endline will examine additional, long-term outcomes of program

participation, including satisfaction with and utilization of facility-based healthcare, family planning utilization, contraception, and infant and child health services such as vaccination. Beyond the current study, research should examine methods to optimize gANC, such as isolating specific services that are most engaging, utilized, and efficacious in promoting maternal and child health behaviors.[23] One possible approach would be to examine if promotion of gANC through channels such as digital media, such as social media or patient-provider apps, would increase program participation and intended outcomes discussed earlier.[24]

CONCLUSION

Implementation of the gANC program at scale in Nigeria produced high levels of participation, and results in terms of facility birthing utilization were consistent with previous research. The program is a promising approach to promoting maternal and child healthcare in LMICs. Future studies should examine long-term effects of the program on participants and ways to optimize gANC implementation.

Acknowledgements: The authors acknowledge the support of the Gates Foundation for this study.

Authors contributions:

Declaration of conflict: The authors have no conflicts of interest to declare.

Funding statement: This study was funded by a grant from the Gates Foundation (INV-043363).

Data access: Access to the data will be provided upon reasonable request submitted to the corresponding author.

References

1. Grenier L, Lori JR, Darney BG, Noguchi LM, Maru S, Klima C, Lundeen T, Walker D, Patil CL, Suhowatsky S, Musange S. Building a Global Evidence Base to Guide Policy and Implementation for Group Antenatal Care in Low-and Middle-Income Countries: Key Principles and Research Framework Recommendations from the Global Group Antenatal Care Collaborative. Journal of midwifery & women's health. 2020 Sep;65(5):694-9.

2. Fagbamigbe AF, Olaseinde O, Setlhare V. Sub-national analysis and determinants of numbers of antenatal care contacts in Nigeria: assessing the compliance with the WHO recommended standard guidelines. BMC Pregnancy and Childbirth. 2021 May 25;21(1):402.

3. Department of Health. Preparation for Birth and Beyond: A resource pack for leaders of community groups and activities.

4. Mehay A, Motta GD, Hunter L, Rayment J, Wiggins M, Haora P, McCourt C, Harden A. What are the mechanisms of effect of group antenatal care? A systematic realist review and synthesis of the literature. BMC pregnancy and childbirth. 2024 Oct 1;24(1):625.

5. Robertson B, Aycock DM, Darnell LA. Comparison of centering pregnancy to traditional care in Hispanic mothers. Maternal and child health journal. 2009 May;13:407-14.

6. Jafari F, Eftekhar H, Mohammad K, Fotouhi A. Does group prenatal care affect satisfaction and prenatal care utilization in Iranian pregnant women?. Iranian journal of public health. 2010 Jun 30;39(2):52.

7. Apetorgbor V, Awini E, Ghosh B, Zielinski R, Amankwah G, Kukula VA, James K, Williams JE, Lori JR, Moyer CA. The impact of group antenatal care on newborns: Results of a cluster randomized control trial in Eastern Region, Ghana. BMC pediatrics. 2024 Nov 18;24(1):747.

8. Kukula VA, Awini E, Ghosh B, Apetorgbor V, Zielinski R, Amankwah G, Ofosu WK, James K, Williams JE, Lori JR, Moyer CA. Effect of group antenatal care versus individualized antenatal care on birth preparedness and complication readiness: a cluster randomized controlled study among pregnant women in Eastern Region of Ghana. BMC Pregnancy and Childbirth. 2024 Aug 16;24(1):546.

9. Jolivet RR, Uttekar BV, O'Connor M, Lakhwani K, Sharma J, Wegner MN. Exploring perceptions of group antenatal Care in Urban India: results of a feasibility study. Reproductive Health. 2018 Dec;15:1-1.

10. Zielinski R, Abdelnabi S, Amankwah G, Kukula VA, Apetorgbor V, Awini E, Williams J, Moyer C, Ghosh B, Lori JR. Knowledge, Acceptance, and Uptake of Family Planning: A Cluster Randomized Controlled Trial of Group Antenatal Care in Ghana. International Journal of Environmental Research and Public Health. 2024 Aug 3;21(8):1025.

11. van Eijk AM, Hill J, Alegana VA, Kirui V, Gething PW, ter Kuile FO, Snow RW. Coverage of malaria protection in pregnant women in sub-Saharan Africa: a synthesis and analysis of national survey data. Lancet Infect Dis. 2011 Mar;11(3):190-207. doi: 10.1016/S1473-3099(10)70295-4. Epub 2011 Jan 26.

12. Lawn JE, Blencowe H, Waiswa P, Amouzou A, Mathers C, Hogan D, Flenady V, Frøen JF, Qureshi ZU, Calderwood C, Shiekh S. Stillbirths: rates, risk factors, and acceleration towards 2030. The Lancet. 2016 Feb 6;387(10018):587-603.

13. Evans WD, Ochu CL, Bingenheimer JB, Adebayo SB, David FA, Gar SA, Abdulkarim M. Implementation and Baseline Evaluation of an Evidence-Based Group Antenatal Care Program in Two Nigerian States. International Journal of Environmental Research and Public Health. 2024 Nov;21(11):1461.

14. Guo S, Fraser MW. Propensity score analysis: Statistical methods and applications. SAGE publications; 2014 Jun 11.

15. Grenier L, Suhowatsky S, Kabue MM, Noguchi LM, Mohan D, Karnad SR, Onguti B, Omanga E, Gichangi A, Wambua J, Waka C. Impact of group antenatal care (G-ANC) versus individual antenatal care (ANC) on quality of care, ANC attendance and facility-based delivery: a pragmatic cluster-randomized controlled trial in Kenya and Nigeria. PLoS One. 2019 Oct 2;14(10):e0222177.

16. Dearing JW, Cox JG. Diffusion of innovations theory, principles, and practice. Health affairs. 2018 Feb 1;37(2):183-90.

17. Carpenter D, Hassell S, Mardon R, Fair S, Johnson M, Siegel S, Nix M. Using learning communities to support adoption of health care innovations. The Joint Commission Journal on Quality and Patient Safety. 2018 Oct 1;44(10):566-73.

18. Grenier L, Onguti B, Whiting-Collins LJ, Omanga E, Suhowatsky S, Winch PJ. Transforming women's and providers' experience of care for improved outcomes: a

theory of change for group antenatal care in Kenya and Nigeria. PLoS One. 2022 May 3;17(5):e0265174.

19. Austin PC, Stuart EA. Moving towards best practice when using inverse probability of treatment weighting (IPTW) using the propensity score to estimate causal treatment effects in observational studies. Stat Med 2015; 34: 3661–3679.

20. Cattaneo MD. Efficient semiparametric estimation of multi-valued treatment effects under ignorability. *Journal of Econometrics.* 2010; 155: 138‑154. https://doi.org/10.1016/j.jeconom.2009.09.023.

21. StataCorp. *Stata Statistical Software: Release 18*. 2023. College Station, TX: StataCorp LLC.

22. Jiwani SS, Amouzou-Aguirre A, Carvajal L, Chou D, Keita Y, Moran AC, Requejo J, Yaya S, Vaz LM, Boerma T. Timing and number of antenatal care contacts in low and middle-income countries: Analysis in the Countdown to 2030 priority countries. J Glob Health. 2020 Jun;10(1):010502. doi: 10.7189/jogh.10.010502.

23. Alem AZ, Yeshaw Y, Liyew AM, Tesema GA, Alamneh TS, Worku MG, Teshale AB, Tessema ZT. Timely initiation of antenatal care and its associated factors among pregnant women in sub-Saharan Africa: A multicountry analysis of Demographic and Health Surveys. PLoS One. 2022 Jan 10;17(1):e0262411. doi: 10.1371/journal.pone.0262411.

24. Till S, Mkhize M, Farao J, Shandu LD, Muthelo L, Coleman TL, Mbombi M, Bopape M, Klingberg S, van Heerden A, Mothiba T, Densmore M, Verdezoto Dias NX; CoMaCH Network. Digital Health Technologies for Maternal and Child Health in Africa and Other Low- and Middle-Income Countries: Cross-disciplinary Scoping Review With

Stakeholder Consultation. J Med Internet Res. 2023 Apr 7;25:e42161. doi:

10.2196/42161.

Tables

Table 1: Distribution of Sociodemographic and Prior Pregnancy- and Delivery-Related Variables among Participants in the Analytic Sample (n=1952)

	N	Percent
State		
Kaduna	906	46.4
Kano	1,046	53.6
Age Group		
15-19	259	13.3
20-24	702	36.0
25-29	498	25.5
30-34	296	15.2
35+	197	10.1
Education		
Never attended formal school	486	24.9
Primary	412	21.1
Secondary	717	36.7
Higher	198	10.1
Qur’anic/Islamiyya	133	6.8
Other	6	0.3
Employment Status		
Unemployed	818	41.9
Employed	101	5.2
Own a business	1,033	52.9
Residence		
Urban	721	36.9
Rural	1,231	63.1
Ever Given Birth Before		
Yes	1,478	75.7
No	474	24.3
Number of Living Children		
0	506	25.9
1	363	18.6
2	301	15.4
3	270	13.8
4	216	11.1
5+	296	15.2
Experienced Problems During Prior Pregnancy/Delivery		
No prior pregnancy/delivery	404	20.7
Yes	516	26.4
No	1,032	52.9
Place of Delivery for Last Pregnancy		

No prior pregnancy/delivery	404	20.7
Home	773	39.6
Facility	736	37.7
Other	39	2.0
Skilled Birth Attended at Last Delivery		
No prior pregnancy/delivery	404	20.7
Yes	990	50.7
No or Don't know	558	28.6
Postnatal Health Check within One Week of Last Delivery		
No prior pregnancy/delivery	404	20.7
Yes	1,273	65.2
No or Don't know	275	14.1
Any Postnatal Care Appointments after Last Delivery		
No prior pregnancy/delivery	404	20.7
Yes	1,326	67.9
No or Don't know	222	11.4

Table 2: Distribution of gANC Meeting Attendance and Facility Delivery (n=1952)

Number of gANC Meetings Attended during Index Pregnancy	n	%
0	180	9.2
1	168	8.6
2	254	13.0
3	367	18.8
4	395	20.2
5	588	30.1
Index Pregnancy Delivered in a Health Facility		
No	1,010	51.7
Yes	942	48.3

Table 3: Associations between Background Variables and Number of Meetings Attended (n=1952)

	Mean	(SD)	p-value
State			0.012
Kaduna	3.73	(1.37)	
Kano	2.79	(1.71)	
Age Group			0.390
15-19	3.05	(1.70)	
20-24	3.28	(1.61)	
25-29	3.33	(1.62)	
30-34	3.11	(1.68)	
35+	3.18	(1.59)	
Education			0.225
Never attended formal school	2.98	(1.67)	
Primary	3.18	(1.64)	
Secondary	3.32	(1.61)	
Higher	3.68	(1.50)	
Qur'anic/Islamiyya	3.06	(1.63)	
Other	3.67	(1.21)	
Employment			0.109
Unemployed	3.06	(1.69)	
Employed	3.77	(1.48)	
Own a business	3.30	(1.58)	
Residence			0.612
Urban	3.36	(1.57)	
Rural	3.15	(1.66)	
Ever Given Birth Before			0.936
Yes	3.22	(1.63)	
No	3.23	(1.64)	
Number of Living Children			0.372
0	3.23	(1.65)	
1	3.25	(1.63)	
2	3.31	(1.70)	
3	3.34	(1.59)	
4	3.13	(1.65)	
5+	3.07	(1.55)	
Experienced Problems During Prior Pregnancy/Delivery			0.149
No prior pregnancy/delivery	3.29	(1.62)	
Yes	3.05	(1.70)	
No	3.28	(1.59)	
Place of Delivery for Last Pregnancy			0.091
No prior pregnancy/delivery	3.29	(1.62)	
Home	3.00	(1.68)	
Facility	3.45	(1.55)	

Other	2.74	(1.71)	
Skilled Birth Attended at Last Delivery			0.234
No prior pregnancy/delivery	3.29	(1.62)	
Yes	3.33	(1.58)	
No or Don't know	2.99	(1.71)	
Postnatal Health Check within One Week of Last Delivery			0.107
No prior pregnancy/delivery	3.29	(1.62)	
Yes	3.27	(1.62)	
No or Don't know	2.94	(1.67)	
Any Postnatal Care Appointments after Last Delivery			0.193
No prior pregnancy/delivery	3.29	(1.62)	
Yes	3.27	(1.61)	
No or Don't know	2.85	(1.74)	
Total	3.23	(1.63)	

Table 4: Associations between Background Variables and Percent Delivering in a Health Facility (n=1952)

	Percent	p-value
State		0.001
Kaduna	64.0	
Kano	34.6	
Age Group		0.252
15-19	47.1	
20-24	52.1	
25-29	45.8	
30-34	46.0	
35+	45.7	
Education		0.000
Never attended formal school	30.9	
Primary	44.7	
Secondary	58.0	
Higher	68.7	
Qur'anic/Islamiyya	39.9	
Other	50.0	
Employment		0.055
Unemployed	46.5	
Employed	60.4	
Own a business	48.5	
Residence		0.000
Urban	64.5	
Rural	38.8	
Ever Given Birth Before		0.009
Yes	45.4	
No	57.2	
Number of Living Children		0.003
0	56.3	
1	51.5	
2	44.2	
3	49.6	
4	41.7	
5+	38.2	
Experienced Problems During Prior Pregnancy/Delivery		0.004
No prior pregnancy/delivery	57.2	
Yes	45.0	
No	46.4	
Place of Delivery for Last Pregnancy		0.000
No prior pregnancy/delivery	57.2	
Home	35.2	
Facility	56.3	

Other	64.1	
Skilled Birth Attended at Last Delivery		0.000
No prior pregnancy/delivery	57.2	
Yes	49.9	
No or Don’t know	38.9	
Postnatal Health Check within One Week of Last Delivery		0.000
No prior pregnancy/delivery	57.2	
Yes	47.0	
No or Don’t know	41.1	
Any Postnatal Care Appointments after Last Delivery		0.000
No prior pregnancy/delivery	57.2	
Yes	46.7	
No or Don’t know	41.4	

Table 5: Unadjusted and Adjusted Associations between Number of gANC Meetings Attended and Delivery in a Health Facility (n=1952)

		Unadjusted		Regression Adjusted		IPW Adjusted	
Number of gANC Meetings Attended	Percent Delivering in a Health Facility	OR	(95% C.I.)	AOR	(95% C.I.)	AOR	(95% C.I.)
0	28.3	0.79	(0.47-1.34)	1.03	(0.62-1.72)	1.11	(0.89-1.33)
1	33.3	1.00	(reference)	1.00	(reference)	1.00	(reference)
2	37.8	1.22	(0.75-1.98)	1.29	(0.77-2.28)	1.06	(0.93-1.18)
3	47.7	1.82	(1.17-2.83)**	1.73	(1.12-2.68)*	1.13	(0.99-1.26)
4	51.4	2.11	(1.34-3.35)**	2.01	(1.33-3.02)**	1.16	(1.03-1.28)**
5	61.4	3.18	(1.60-6.33)**	2.73	(1.45-5.14)**	1.24	(1.04-1.44)**

Supplementary files

Click here to access/download
Supporting Information
IRB Approval_gANC_Kaduna and Kano States.pdf

Click here to access/download
Supporting Information
IRB determination_WDE_gANC.pdf
