# Supplementary material for: Evaluation of a group antenatal care intervention in two Northern Nigerian states: Quasi-experimental study
Source: PLoS One. 2025 Oct 8;20(10):e0333383. doi: 10.1371/journal.pone.0333383 (PMC12507231; doi:10.1371/journal.pone.0333383)
Supplement: Attachment — Submitted filename: Response_memo_30August2025.docx [file pone.0333383.s003.docx]

RESPONSE TO REVIEWERS:

In the following, we respond point-by-point to the four reviewers’ comments. We have edited the resubmitted manuscript and highlighted all new and revised text and materials in yellow and in teal. In addition, we have responded to all of the editorial office comments in this resubmission.

Reviewer #1: comments
¬ The introduction provides a comprehensive overview of the rationale for group antenatal care (gANC) in LMICs, especially Nigeria, and summarizes relevant evidence. However, it would benefit from a clearer articulation of what this specific study adds to the existing literature. For example, explicitly state how this is the first large-scale, quasi-experimental, longitudinal evaluation of gANC in Kaduna and Kano, and how its midline findings fill a gap in knowledge about real-world implementation and effectiveness in these settings

AUTHOR RESPONSE: WE THANK THE REVIEWER AND HAVE EDITED THE INTRODUCTION ACCORDINGLY.

¬ The introduction mentions high non-utilization rates of ANC in northern Nigeria but could more directly link this to the choice of Kaduna and Kano as study sites. Briefly highlight the distinct demographic, socioeconomic, and cultural factors in these states that make them both representative and challenging for ANC interventions

AUTHOR RESPONSE: WITHIN SPACE CONSTRAINTS, WE HAVE ADDED DETAILS ON THESE FACTORS.

¬ Use consistent terminology for group antenatal care (gANC or G-ANC) and individual antenatal care (I-ANC) throughout the introduction.

AUTHOR RESPONSE: WE HAVE MADE THIS CORRECTION.

¬ Provide more detail on the sampling strategy: How were healthcare facilities and participants selected? Was randomization used at any stage? Clarify inclusion and exclusion criteria, and discuss any potential for selection bias

AUTHOR RESPONSE: WE HAVE ADDED DETAILS ON THE SAMPLING PLAN TO THE METHODS SECTION.

¬ The section describes the use of a 154-item questionnaire and fieldwork procedures, but could be strengthened by detailing: How interviewers were trained and monitored, How data quality was assured during fieldwork, Any steps taken to minimize interviewer bias or respondent misunderstanding

AUTHOR RESPONSE: WITHIN SPACE CONSTRAINTS, WE DESCRIBED THE INTERVIEWER TRAINING AND QUALITY CONTROL.
¬ The methods should specify how missing data were addressed in the analysis (e.g., imputation, exclusion, sensitivity analyses), especially given the longitudinal design and potential for attrition

AUTHOR RESPONSE: WE HAVE ADDED A CLAUSE TO THE FIRST SENTENCE OF THE FIRST PARAGRAPH OF DATA ANALYSIS, PLUS AN ENTIRE SENTENCE AT THE END OF THAT PARAGRAPH, AND AN APPENDIX TABLE SPECIFYING THAT WE CONDUCTED COMPLETE-CASE ANALYSIS AND JUSTIFYING THAT APPROACH.

¬ Describe how data for intervention (gANC) and comparison (I-ANC) groups were stored separately (e.g., encrypted databases with unique identifiers) to prevent contamination.

AUTHOR RESPONSE: WE NOTE THAT THIS STUDY ONLY EXAMINED THE gANC PROGRAM. WE HAVE ADDED DETAIL ON DATA MANAGEMENT AND STORAGE.

¬ Emphasize the significance of the high retention (87.1%) and attendance rates in the context of known challenges with ANC utilization in northern Nigeria, highlighting how this supports the feasibility and acceptability of gANC in these settings.

AUTHOR RESPONSE: WE HAVE EMPHASIZED THESE POINTS.

¬ Provide more detail on what aspects of the gANC implementation contributed to high attendance and retention (e.g., community mobilization, group dynamics, provider engagement), referencing qualitative findings if available.

AUTHOR RESPONSE: WHILE WE AGREE THAT THIS IS IMPORTANT FOR FUTURE RESEARCH, THESE DETAILS ARE BEYOND THE SCOPE OF OUR DATA AND THE CURRENT STUDY.

¬ Mention any challenges encountered during implementation and how they were addressed or could be mitigated in future scale-up.

AUTHOR RESPONSE: WE AGREE THAT THESE ARE IMPORTANT CONSIDERATIONS, BUT THIS IS BEYOND THE SCOPE OF OUR CURRENT STUDY. WE HAVE NOTED THE IMPORTANCE OF STUDYING IMPLEMENTATION IN THE DISCUSSION.

¬ Acknowledge the lack of randomization and potential for residual confounding despite inverse probability weighting.

AUTHOR RESPONSE: WE HAVE NOTED THIS AS A LIMITATION IN THE DISCUSSION.

Reviewer #2: Comments:

1. Lack of Clarity and Specificity in Background:
The background is overly generic and fails to clearly establish the research gap or the rationale for using gANC in the Nigerian context. It references high ANC non-utilization in "some parts of Nigeria" without citing specific statistics, regions, or studies.

The link between gANC and improved maternal outcomes is claimed without sufficient supporting literature. Assertions such as “gANC has the potential to reshape traditional ANC” are unsubstantiated and speculative.

AUTHOR RESPONSE: WITHIN SPACE CONSTRAINTS, WE HAVE ADDED DETAILS TO THE BACKGROUND PER THESE COMMENTS AND THOSE OF OTHER REVIEWERS.

No theoretical framework or conceptual model underpins the study’s hypothesis.

AUTHOR RESPONSE: THE STUDY IS BASED ON A THEORETICAL FRAMEWORK, WHICH WAS REPORTED IN OUR PREVIOUS PAPER. WE HAVE CLARIFIED THIS POINT AND ADDED DETAILS IN THE METHODS WHERE THE INTERVENTION IS DESCRIBED.

2. Weak and Ambiguous Methodology:
The study design lacks sufficient detail. It is described as "longitudinal," yet no timeline, follow-up intervals, or baseline characteristics are presented.

It is unclear how participants were recruited, how exposure to gANC was defined, or what specific inclusion/exclusion criteria were used.

The use of inverse probability weighting and propensity score matching is stated, but the manuscript does not describe the model diagnostics, variable selection process, or balance statistics. This undermines the validity of the causal claims.

There is no reporting of confidence intervals, p-values, or model fit statistics. The manuscript vaguely claims "positive association" without statistical evidence.

AUTHOR RESPONSE: WE HAVE ADDED DETAILS TO THE METHODS SECTION TO ADDRESS EACH OF THESE POINTS AS NECESSARY AND APPROPRIATE NOTE THAT THE TIMELINE FOR BASELINE AND FOLLOW-UP DATA COLLECTION IS DELINEATED IN THE FIRST PARAGRAPH OF THE DATA COLLECTION SUBSECTION OF THE METHODS SECTION; THE OPERATIONAL DEFINITION OF GANC EXPOSURE IS DESCRIBED IN DETAIL IN THE FIRST PARAGRAPH OF THE MEASURES SUBSECTION OF THE METHODS SECTION; TABLES 3 AND 4 INCLUDE P-VALUES WHILE TABLE 5 INCLUDES CONFIDENCE INTERVALS AND ASTERISKS INDICATING LEVELS OF STATISTICAL SIGNIFICANCE. WE HAVE ADDED TWO SUPPLEMENTARY TABLES (NUMBERS S2 and S3) PROVIDING RESULTS OF THE MULTINOMIAL LOGISTIC REGRESSION MODEL THAT FORMED PART OF THE INVERSE-PROBABILITY WEIGHTING ANALYSIS AND UNWEIGHTED AND WEIGHTED BALANCE STATISTICS FOR THE MODEL COVARIATES ALONG WITH SOME ACCOMPANYING TEXT.

3. Results Are Descriptive and Lack Analytical Depth:
The results section reads like a narrative summary and fails to provide tabulated data, model outputs, or measures of uncertainty.

There is no stratification by relevant subgroups (e.g., rural vs. urban, parity, age).

Terms like "high attendance" and "low attrition" are vague. The reader is not told how many participants were recruited, followed up, or lost, nor how attrition may have biased the results.

AUTHOR RESPONSE: WE RESPECTFULLY NOTE THAT THE ORIGINAL MANUSCRIPT INCLUDED FIVE TABLES, TWO OF WHICH INCLUDED P-VALUES AND ONE OF WHICH INCLUDED CONFIDENCE INTERVALS. MULTIVARIATE ANALYSES WERE PRESENTED, AND CO-VARIATES FOR RELEVANT SUBGROUPS WERE INCLUDED, AS DESCRIBED IN THE METHODS AND RESULTS. WE HAVE CLARIFIED THESE POINTS IN THE REVISION. THE EXTENT OF ATTRITION / RETENTION IS DELINEATED QUANTITATIVELY IN THE FIRST PARAGRAPH OF THE RESULTS SECTION.

4. Unsupported Causal Claims:
The manuscript repeatedly implies causality (“causal effect of gANC on facility delivery”) despite relying on observational data.

While statistical methods such as IPW are mentioned, the paper does not demonstrate their correct application or justify assumptions necessary for causal inference (e.g., no unmeasured confounding, positivity, consistency).

AUTHOR RESPONSE: A FAIRLY ROBUST DESCRIPTION OF THE INVERSE-PROBABILITY WEIGHTING ANALYSIS APPEARS IN THE LAST TWO PARAGRAPH OF THE DATA ANALYSIS SUBSECTION OF THE METHODS SECTION OF THE MANUSCRIPT. WE HAVE ADDED TWO SUPPLEMENTARY TABLES (S1 AND S2) THAT FURTHER DELINEATE THE SPECIFICATION OF THE IPW MODEL AND RESULTS THEREOF, INCLUDING AN ANALYSIS OF COVARIATE BALANCE (Table S3). WITH RESPECT TO THE ISSUE OF CAUSAL INFERENCE, THE ENTIRE SECOND PARAGRAPH OF THE LIMITATIONS SECTION IS DEVOTED TO THE POSSIBILITY OF OMITTED VARIABLE BIAS.

5. Poor Structure and Repetitive Content:
There is repetition of key points across sections (e.g., that attendance is associated with facility delivery is stated in background, results, discussion, and conclusion without added nuance).

AUTHOR RESPONSE: WE HAVE EDITED TO REDUCE REPETITION.

The writing is vague, non-scientific, and sometimes speculative, e.g., "scalable in diverse urban and rural settings" is stated without scalability analysis or implementation details.

There is a lack of clear outcome definitions—e.g., how was facility delivery validated or measured?

AUTHOR RESPONSE: WE HAVE EDITED THE OUTCOMES STATEMENTS AND ENSURED THAT ALL CLAIMS ARE VALIDATED.

6. No Contribution to Existing Literature:
The manuscript claims its results confirm prior findings but does not offer new insights, mechanisms, or innovations in delivery or evaluation of gANC.

There is no critical discussion of potential biases, limitations, or alternative explanations.

AUTHOR RESPONSE: WE RESPECTFULLY NOTE THAT THIS IS THE FIRST STUDY OF ITS KIND, AND MAKES A NOVEL CONTRIBUTION TO THE LITERATURE. FURTHER, IT VALIDATES A PREVIOUS RANDOMIZED CONTROLLED TRIAL IN A LARGE-SCALE, FIELD-BASED SETTING. WE HAVE EMPHASIZED THESE POINTS IN THE REVISION.

7. Ethical Considerations and Data Transparency Missing:
No mention of ethical approval, informed consent, or data protection procedures for participants in a longitudinal cohort study.

No indication of data availability, data sharing policies, or trial registration if applicable.

AUTHOR RESPONSE: THESE POINTS WERE PROVIDED IN THE META-DATA, AND WE HAVE EDITED THE MANUSCRIPT TO ENSURE CLARIFY REGARDING ETHICAL APPROVAL AND DATA AVAILABILITY.

Reviewer #3: Dear Author,

Thank you for this study which is a good addition to antenatal care in Northern Nigeria.
I see this study as a simplified form of antenatal care with focus on improving maternal and child health outcomes.
Please attend to my concerns below:

TITLE: This title needs to be reviewed. I think the title should reflect the focus of the study more than the methodology. It should also be minimally encompassing and reflects the fraction of the study area as much as possible. suggested title would be like: Group Antenatal Care Intervention in Two Northern Nigerian states: A Quasi-experimental evaluation/study/assessment.

AUTHOR RESPONSE: THANK YOU, WE HAVE EDITED THE TITLE ALONG THE LINES SUGGESTED.

ABSTRACT: Good abstract. Clear and concise, well focused and informative. Thank you.
You may need to include the "keywords"

AUTHOR RESPONSE: WE HAVE ENSURED THAT THE KEYWORDS ARE INCLUDED (THEY WERE IN THE META-DATA). THANK YOU.

INTRODUCTION: Generally, this introduction meets the necessary requirement for the follow up phase of a longitudinal study. The social and scientific values of the study are well presented. Thank you.

Paragraph2: It is generic that standard/focused antenatal education improves maternal and fetal outcomes in any pregnancy. The level of influence/impact may be debatable and subject to other independent variables.
Please rephrase this statement.

AUTHOR RESPONSE: WE HAVE REPHRASED AS SUGGESTED. THANK YOU.

MEASURES:

P1: You meant " presenting problems"?
P2: Measurement of this dependent variable would definitely reflect the successful impact of gANC to influence antenatal health seeking behaviors, however, i think measuring the pregnancy outcomes would be of equal clinical significance,
Consider adding this if data is available.

AUTHOR RESPONSE: WE HAVE EDITED FOR THESE SPECIFIC POINTS. WE NOTE THAT PREGNANCY OUTCOME (BIRTH IN A FACILITY) WAS THE PRIMARY ENDPOINT.

RESULTS:
P1: please use a uniform numerical representation: either x, y% or x (y%). Thanks

AUTHOR RESPONSE: CHANGE MADE.

P1: Is this a pregnancy related death? Clarify if known.

AUTHOR RESPONSE: YES, AND WE HAVE CLARIFIED.

TABLE2: This is very important in this study and also remarkable for a longitudinal study. Ability to shift this upwardly will determine the clinical and statistical impact of this study. Thank

AUTHOR RESPONSE: WE THANK THE REVIEWER FOR THIS COMMENT.

FUTURE RESEARCH: I think this study needs to consider a mixed research method for the 3rd series of the study. I think a semi-structured or focus group interview of some of the participants will answer a lot of questions that will strengthen the goal of the study.

AUTHOR RESPONSE: THIS IS BEYOND THE SCOPE OF THE CURRENT STUDY, BUT WE HAVE NOTED THE IMPORTANCE OF MIXED METHODS AND PLANS TO CONDUCT SUCH ANALYSES IN A FUTURE MANUSCRIPT/STUDY.

Reviewer #4: The authors have presented their work with sound argument and discuss their findings with rigour. However, because this is a follow up write up of a larger work it would have helped if they summarised earlier findings of the work to give context frothier findings in this work. This does not take away from the work presented but it could have improved on the presentation. This said, thank you for the opportunity to review this work.
The work is significant as maternal mortality remains a challenge in LMICs. Increasing coverage and improving affordability through gANC will go a long way if implemented adequately. I believe this work can be published as is.

AUTHOR RESPONSE: WE THANK THE REVIEWER FOR THESE SUPPORTIVE COMMENTS. MUCH APPRECIATED.
