# Supplementary material for: Evaluation of a group antenatal care intervention in two Northern Nigerian states: Quasi-experimental study
Source: PLoS One. 2025 Oct 8;20(10):e0333383. doi: 10.1371/journal.pone.0333383 (PMC12507231; doi:10.1371/journal.pone.0333383)
Supplement: Attachment — Submitted filename: Response memo_10Sept2025.docx [file pone.0333383.s004.docx]

**RESPONSE TO REVIEWERS**

You mentioned in the manuscript that "**In terms of number of gANC sessions attended, the distribution is skewed toward higher values, with five sessions being the modal value, followed by four sessions, and so on.**" If this variable is not normally distributed, why did you not report it's estimates in median instead of mean; and why did you choose to apply parametric test for its bivariate association with the covariates in Table 3?

Thank you for this question. We elected to use a parametric approach here for two reasons. First and foremost, we wanted our statistical inferences (specifically, p-values) to reflect the clustering of individual participants within local government areas (LGAs). This can be done in several ways in parametric analyses, but not, to our knowledge, in non-parametric analyses. Our approach was to treat number of meanings as a continuous dependent variable and model it in relation to the covariates using linear regression with the Stata option vce(cluster w0_bmgf_lga) specified. This results on robust / clustered standard errors and p-values adjusted accordingly. The second reason we selected this approach is that parametric models including linear regression are widely understood by statisticians to be robust to violations of normality assumptions when the sample size is large (as it is here; n=1952), and because parametric tests usually provide greater statistical power than their non-parametric analogs. We could add medians to Table 3, as well as p-values derived from non-parametric tests and are certainly willing to do so. But we believe this is unnecessary and could present a distraction from the main focus of the manuscript, which is the relationship between number of gANC meetings attended and facility delivery.

I would like you to include a brief statement on this in the **Data Analysis** section after this statement: "**We next examine the associations between each of the twelve covariates and number of gANC meetings attended by obtaining the mean and standard deviation of number of sessions attended within each level of each covariate**"

We thank the reviewer for this suggestion and have added the following sentence explaining our reasoning: “This approach is justified in spite of the skewness of the gANC meeting attendance variable because inference in linear regression is robust to moderate violations of normality assumptions, and because this facilitates the incorporation of clustering of participants within local government authorities as described below.”
